# Heterologous expression of CTP:phosphocholine cytidylyltransferase from *Plasmodium falciparum* rescues Chinese Hamster Ovary cells deficient in the Kennedy phosphatidylcholine biosynthesis pathway

**DOI:** 10.1038/s41598-018-27183-w

**Published:** 2018-06-12

**Authors:** Lívia Marton, Fanni Hajdú, Gergely N. Nagy, Nóra Kucsma, Gergely Szakács, Beáta G. Vértessy

**Affiliations:** 1 Institute of Enzymology, Research Centre for Natural Sciences, Hungarian Academy of Sciences Budapest, 1117 Hungary; 2 Doctoral School of Multidisciplinary Medical Science, University of Szeged, Szeged, 6720 Hungary; 3 Department of Applied Biotechnology and Food Science, Budapest University of Technology and Economics, Budapest, 1111 Hungary; 4 Present Address: Division of Structural Biology, University of Oxford, Roosevelt Drive, Oxford, OX37BN United Kingdom; 5 Present Address: Institute of Cancer Research, Medical University Vienna, Vienna, Austria

## Abstract

The plasmodial CTP:phosphocholine cytidylyltransferase (*Pf*CCT) is a promising antimalarial target, which can be inhibited to exploit the need for increased lipid biosynthesis during the erythrocytic life stage of *Plasmodium falciparum*. Notable structural and regulatory differences of plasmodial and mammalian CCTs offer the possibility to develop species-specific inhibitors. The aim of this study was to use CHO-MT58 cells expressing a temperature-sensitive mutant CCT for the functional characterization of *Pf*CCT. We show that heterologous expression of wild type *Pf*CCT restores the viability of CHO-MT58 cells at non-permissive (40 °C) temperatures, whereas catalytically perturbed or structurally destabilized *Pf*CCT variants fail to provide rescue. Detailed *in vitro* characterization indicates that the H630N mutation diminishes the catalytic rate constant of *Pf*CCT. The flow cytometry-based rescue assay provides a quantitative readout of the *Pf*CCT function opening the possibility for the functional analysis of *Pf*CCT and the high throughput screening of antimalarial compounds targeting plasmodial CCT.

## Introduction

Malaria is still one of the most serious vector-borne infectious diseases threatening approximately 2.75 billion people worldwide^1^. The deadliest form of the disease is caused by the protozoan parasite *Plasmodium falciparum*. Successful attempts for producing a malaria vaccine have been reported recently^2^, however, there is still a need for drug treatments. Widespread drug resistance even against artemisinin-based combination therapies^3^ urges the development of novel antimalarial drugs for the infected patients. Based on the observation that phosphatidylcholine (PC) is the most abundant phospholipid in Plasmodia infected red blood cells^4,5^, the cytidine diphosphate choline (CDP-choline) or Kennedy pathway responsible for *de novo* PC biosynthesis in eukaryotic cells was proposed to be a pharmacological target for the treatment of malaria (see^6^ for a detailed review). Choline as a precursor is converted in three steps to PC by the consecutive action of choline kinase (CK), CTP:phosphocholine cytidylyltransferase (CCT) and choline/ethanolamine phosphotransferase (CEPT) enzymes, with a rate limiting step being catalyzed by CCT. The bis-thiazolium compound albitiazolium was shown to exert its antimalarial activity by inhibiting choline transport and the Kennedy PC biosynthesis enzymes of the parasite^6^. Notably, this pathway was shown to be refractory to genetic disruption, suggesting that it is essential for parasite survival^7^. Lipid biosynthesis and thus CCT was also found to be essential for a large variety of additional organisms indicated by gene disruption or knock-out experiments in organisms or cell lines^8–14^.

CHO-MT58 is a Chinese Hamster Ovarian (CHO) cell line generated by chemical mutagenesis from CHO-K1^14^. In CHO-MT58, the *cct* gene contains a point mutation resulting in an amino acid change (R140H) that deteriorates catalytic domain dimer formation^15^. While at 37 °C (permissive temperature) functional protein is formed albeit in smaller amount, at 40 °C (non-permissive temperature) an accelerated rate of CCT degradation is observed as a result of the point mutation. This temperature induced deficiency causes drastically decreased PC levels, morphological changes such as endoplasmic reticulum (ER) dilation^16^ and eventually leads to apoptosis within 30–48 hours^17^. This cellular model has been extensively used to study the relation of CCT deficiency and apoptosis^16,18–21^. As CHO-MT58 can be rescued either *via* exogenous PC supply, reverting the temperature to 37 °C, or heterologous CCT expression, this cell line has the potential for functional characterization of CCT orthologues in a cellular environment. Considering that at the non-permissive temperature (40 °C) the hamster CCT is not functional, heterologously expressed CCT constructs can be studied with conditional exclusion of the effect of the endogenous thermo-sensitive CCT.

As compared to CCTs, *Plasmodium falciparum* CTP:phosphocholine cytidylyltransferase (*Pf*CCT) displays altered structural organization^22^ and sensitivity to lipid-induced modulation^23^ and thus may be considered as a plausible target for *Plasmodium*-specific drug design. As a result of a gene duplication event, *Pf*CCT is a pseudoheterodimer, containing two highly similar catalytic/membrane binding domains linked with a 305 amino acid long segment^22^. The membrane binding domain of the enzyme has a decisive role in the self-regulatory mechanism of CCT. Still, the auto-inhibition mechanism of *Pf*CCT may differ from that observed for rat CCT (*Rattus norvegicus* CCT, *Rn*CCT) as it was shown that upon membrane binding *Pf*CCT activity increases at most up to 5-fold^24^ (cf. 200-fold in case of *Rn*CCT^25^). The long term aim in our laboratory is to establish a model system to study the *Pf*CCT enzyme function and to build a suitable system for cellular studies of antimalarials targeting *Pf*CCT. Here we report the successful heterologous expression of *Pf*CCT in mammalian CHO cells. We demonstrate that the expression of *Pf*CCT is able to rescue CHO-MT58 cells from temperature-induced apoptosis. We propose an easy-to-implement cellular assay for the functional characterization of *Pf*CCT.

## Results

### Heterologously expressed *Pf*CCT is able to rescue CHO-MT58 cells from apoptosis at the non-permissive temperature

**Figure 1:**
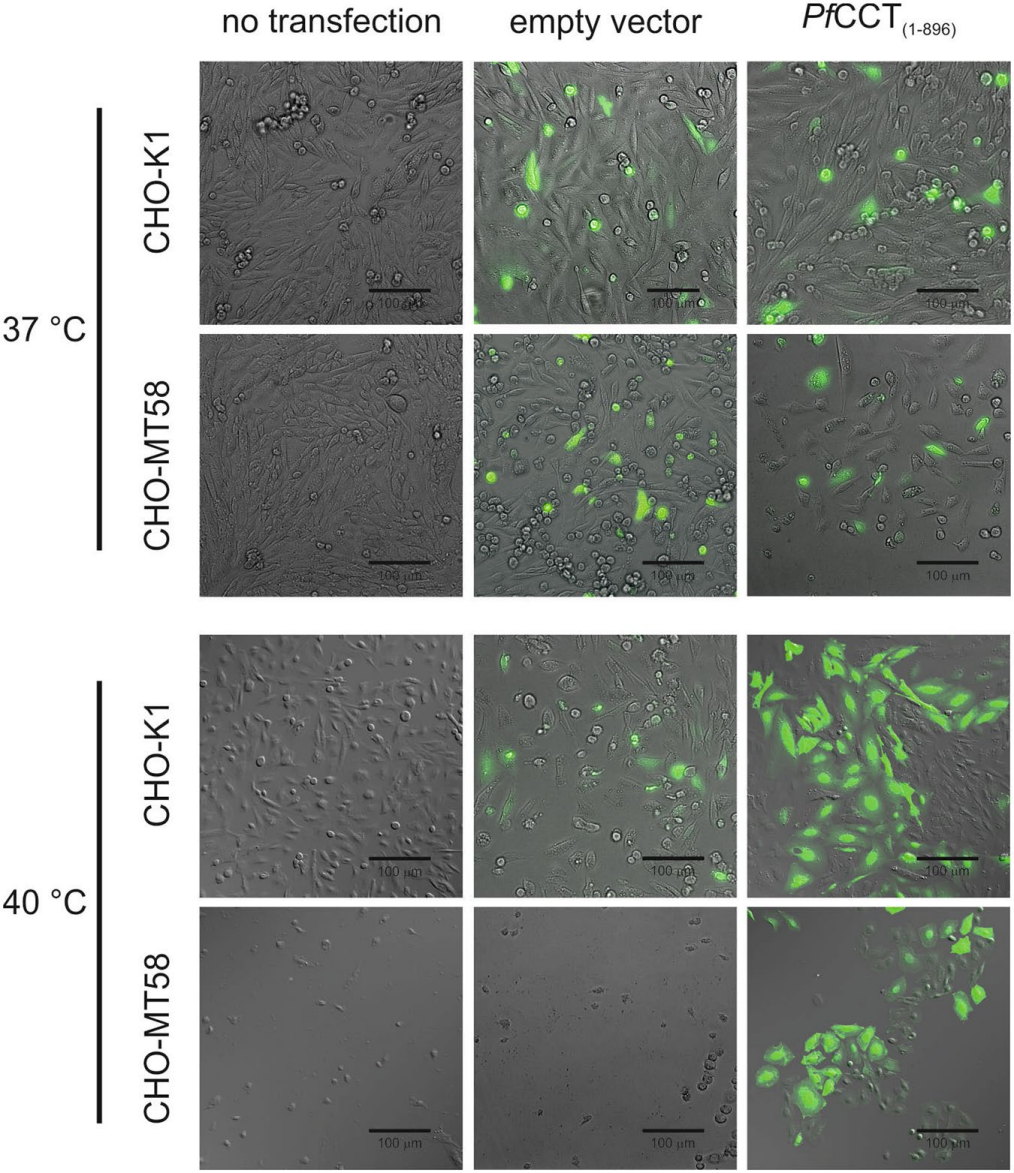
Overexpression of *Pf*CCT_(1–896)_ in CHO cells. Brightfield or DIC and fluorescence microscopic images (40× magnification) were recorded for non-transfected control (left), empty vectot control (middle) and *Pf*CCT/EGFP-transfected (right) CHO-MT58 and CHO-K1 cells (see Materials and Methods) incubated at 37 or 40 °C for 10 days post-transfection. Overlay images were taken from representative areas of the culturing wells.

It has been previously reported that the temperature-induced apoptosis of CHO-MT58 cells^17^ can be rescued by the heterologous expression of CTP:phosphocholine cytidylyltransferase from rat liver^26^. Our aim was to test whether expression of *Pf*CCT can also prevent apoptosis of CHO-MT58 cells cultured at a non-permissive temperature. CHO-K1 and CHO-MT58 cells were transiently transfected with an IRES construct encoding the full length *Pf*CCT_(1–896)_ linked to EGFP (hereafter referred to as *Pf*CCT/EGFP). 24 h post-transfection the temperature was shifted from 37 °C to 40 °C. As reported earlier, the shift to a non-permissive temperature resulted in the apoptosis of CHO-MT58 cells, whereas the proliferation of wild type CHO-K1 cells increased as expected due to shortened cell cycle time at higher temperatures^27^. Figure 1 shows the brightfield or differential interference contrast (DIC) and fluorescence images of control and *Pf*CCT-transfected cells taken 10 days after incubating the cells at 37 °C or shifting the temperature to 40 °C. The overlay images show that the cells incubated at 37 °C all seemed viable. Whereas non-transfected and empty vector transfected CHO-MT58 cells were almost without exception dead after the 10 days 40 °C incubation, wild type CHO-CK1 cells were almost fully confluent. Significantly, a noteworthy proportion of the CHO-MT58 cells could be rescued by the heterologous expression of *Pf*CCT_(1–896)_.

### *In vitro* characterization of an inactive *Pf*CCT mutant for negative control

**Figure 2:**
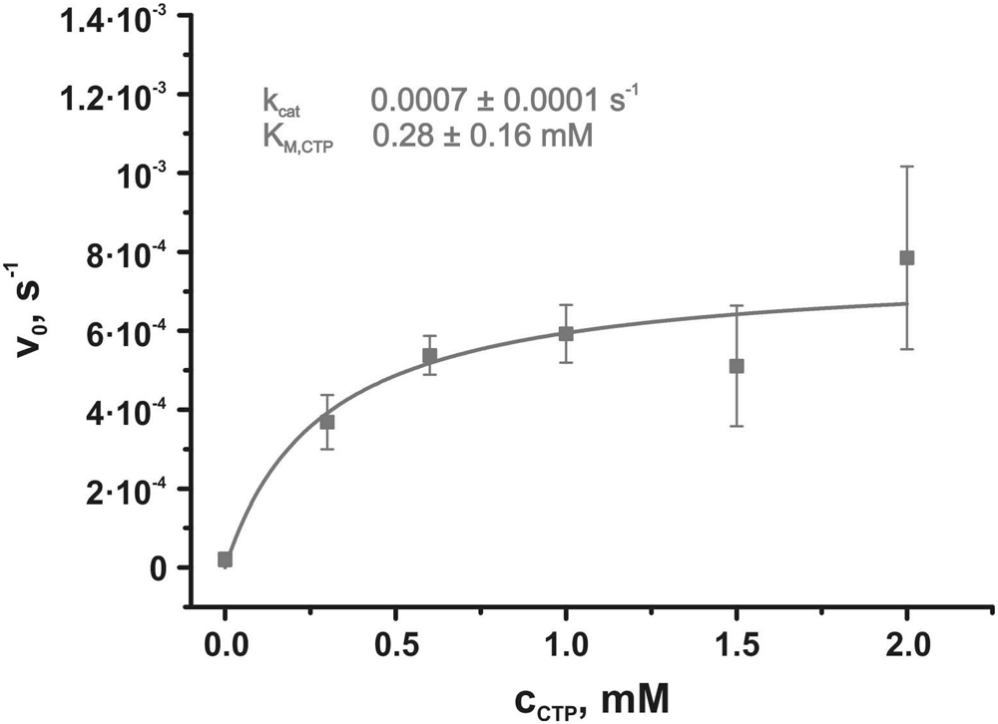
Kinetic analysis of the inactive *Pf*CCT catalytic domain construct. CTP titration of the activity of the inactive *Pf*CCT construct consisting of the second catalytic domain at a fixed concentration of ChoP (5 mM). Data is fitted with the Michaelis-Menten kinetic model assuming no cooperativity. Error bars represent standard deviation from the mean of at least three independent experiments.

Having detected the rescue of CHO-MT58 cells by *Pf*CCT, our next aim was to establish an assay to quantify efficiency. As a control we engineered point mutations to *Pf*CCT corresponding to the R140H thermosensitizing mutation of the endogenous CCT of CHO-MT58^26^. Since the catalytic domain is duplicated in *Plasmodium* CCT^22^, the Arg/His mutation was introduced at the respective positions of residue numbers 96 and 681 in the N-terminal and C-terminal halves of the protein, respectively. We have previously characterized this Arg/His point mutation within the second catalytic domain *in vitro* and demonstrated that the fold and the function of the protein is not damaged, whereas, thermal stability and dimer formation are heavily impaired^15^. To further prove that the rescue is linked to the enzymatic function of CCT, we engineered a *Pf*CCT variant harboring a mutation in the conserved HxGH motif, a unifying feature of the cytidylyltransferase superfamily which has been shown to function in substrate binding and catalysis^28^. In the close relative *Bacillus subtilis* CTP:glycerol-3-phosphate cytidylyltransferase (*Bs*GCT) and the ortholog *Rn*CCT the substitution of the first histidine to alanine (H14A and H89A, respectively) abolishes activity^28,29^ (Supplementary Figure 1). In *Rn*CCT, exchange of the first histidine to asparagine (H89N) had a drastic effect on enzyme catalytic turnover without perturbing CTP binding. To investigate the effect of the mutation corresponding to H89N in *Rn*CCT, a point mutation in the well characterized second catalytic domain of *Pf*CCT_(528–795)_ was generated (H630N). Our *in vitro* kinetic analysis of *Pf*CCT_(528–795)_ H630N mutant revealed that this mutation rendered the enzyme practically inactive since the k_cat_ value was determined to be 0.0007 ± 0.0001 s^−1^. As we showed earlier the wild type enzyme has a k_cat_ value of 1.45 ± 0.05 s^−1 ^^22^, which is over 2000-fold higher than the k_cat_ measured here for the mutant enzyme catalytic activity. On the other hand the K_M,CTP_ was not severely attenuated (0.28 ± 0.16 mM to be compared with the previously determined 0.17 ± 0.02 mM in case of *Pf*CCT_(528–795)_ H630N and *Pf*CCT_(528–795)_^22^, respectively) (Fig. 2). Thus, replacement of the first histidine in the HxGH signature sequence by asparagine in both catalytic domains (H45N and H630N) will yield a catalytically deficient form of *Pf*CCT that corresponds to an inactive enzyme phenotype^30,31^.

### Analysis of *Pf*CCT rescue potential in CHO cells

**Figure 3:**
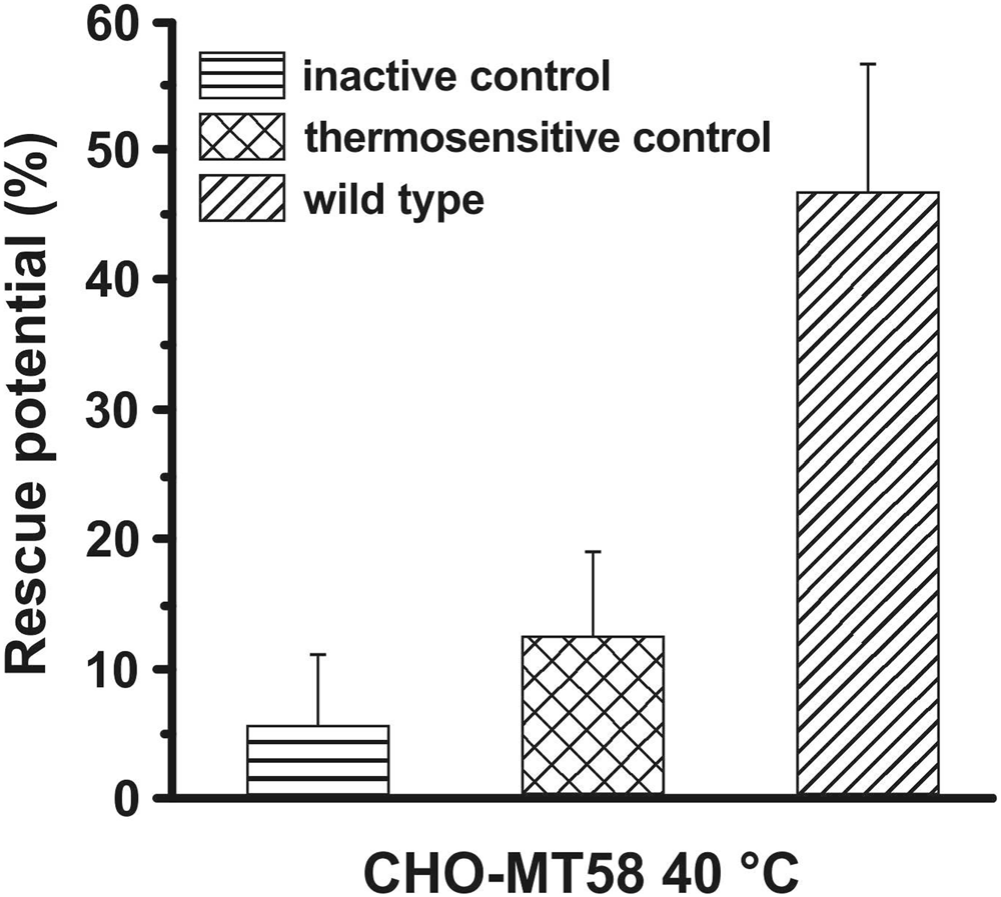
Rescue potential of different *Pf*CCT protein constructs in CHO-MT58 cells at 40 °C. Percentage of rescue potential is determined by flow cytometry experiments based on the proportion of propidium-iodide negative and all cells compared to the control experiments. The wild type column represents the rescue potential of the *Pf*CCT enzyme. The inactive and thermosensitive controls are the *Pf*CCT_(1–896)_ H45N H630N and *Pf*CCT_(1–896)_ R98H R681H double point mutant constructs of the full length *Pf*CCT, respectively. Error bars represent standard deviation from the mean of at least three independent experiments.

Following the kinetic analysis of the enzymatic activity, we next characterized the rescue potential of mutant *Pf*CCT variants in the mammalian CHO model described above. We employed flow cytometry as a suitable technique for the quantitative characterization of heterogeneous cell populations based on differences in fluorescence as well as cell size and cell volume. Full length *Pf*CCT_(1–896)_ and both EGFP-labelled mutant variants (the inactive *Pf*CCT_(1–896)_ H45N H630N double mutant and the thermosensitive *Pf*CCT_(1–896)_ R96H R681H double mutant), were transiently expressed in CHO-K1 and CHO-MT58 cell lines using an internal ribosomal entry site (IRES) system to ensure co-expression of EGFP and *Pf*CCT. Transiently transfected cells were incubated at a permissive temperature (37 °C) for 24 h then the temperature was shifted to 40 °C. Samples were prepared for flow cytometry analysis 72 h post-transfection. The rescue potential of the different *Pf*CCT constructs was calculated based on the proportion of live cells in relation to the corresponding control experiments (Equation (1)). Transfection with the inactive *Pf*CCT_(1–896)_ H45N H630N double mutant construct yielded a rescue potential of 5.6 ± 5.6%. A slightly higher percentage of CHO-MT58 cells (12.4 ± 6.6%) was rescued by the transfection of the thermosensitive *Pf*CCT_(1–896)_ R96H R681H, while 46.7 ± 9.9% of cells expressing the fully functional *Pf*CCT_(1–896)_ were able to escape apoptosis (Table 1, Fig. 3). Although the error of the rescue potential is compromised possibly due to variations in transfection efficiency, the increase in the rescue potential is remarkable and demonstrates that a catalytically functional and structurally intact form of *Pf*CCT is required to rescue CHO-MT58 cells.

**Table 1:** Effect of the overexpression of *Pf*CCT protein variants on the fraction of live CHO-MT58 cells cultured at 40 °C.

*Pf*CCT protein variants	CHO-MT58		
	inactive	thermosensitive	wild type
Live cell fraction (%) at 37 °C	69.5 ± 9.2	77.0 ± 10.2	66.0 ± 13.2
Live cell fraction(%) at 40 °C	3.6 ± 3.4	9.8 ± 5.9	30.3 ± 6.2

Live fraction of cells was detected based on PI staining and intact cell detection. Flow cytometric data is a result of at least three independent experiments. See Supplementary Table 1 for heterologous expression effects on the control CHO-K1 cell line.

## Discussion

*Pf*CCT is the rate-limiting enzyme of the lipid biosynthesis pathway, which contains several validated antimalarial target enzymes^6,22,30,32–34^. The structural and functional differences of *Pf*CCT as compared to *Rn*CCT^23,24,32^ makes it an interesting and relevant enzyme to study in detail, especially regarding its target potential for developing novel antimalarial drugs. However, it has been difficult to investigate the full length protein due to problems in expression and purification. To our best knowledge, this is the first time that functional investigation of the full length *Pf*CCT_(1–896)_ (PF3D7_1316600) entirely encompassing both duplicated domains^22^ is presented. Our results demonstrate that the heterologous expression of full length *Pf*CCT can prevent apoptosis of CHO-MT58 cells under non-permissive hyperthermic conditions (40 °C). We verified that the enzymatic activity of *Pf*CCT is responsible for this rescue effect as expression of a catalytically deficient *Pf*CCT H45N, H630N double mutant yielded considerably lower survival. After the rescue potential of *Pf*CCT was verified, we analyzed the possibility to design a test system using the isogenic CHO-K1 and CHO-MT58 cell lines. CHO cell lines have rapid doubling times and are also resilient to manipulation therefore they are compatible with large-scale studies^35^. Understanding the mechanism underlying the apoptosis induction and the detailed consequences of temperature-induced CCT decay is a necessary pre-requisite for further harnessing this system as a model platform. According to our current knowledge apoptosis due to PC deficiency occurs through the unfolded protein response (UPR) pathways^20,21^. PC depletion activates UPR mainly by an indirect perturbation of the protein-folding environment. Moderate stress initiates *inter alia* ER biogenesis via upregulation of lipid biosynthetic enzymes and ER associated degradation of mis- or unfolded proteins^36^. As in CCT deficient CHO-MT58 cells misfolded dysfunctional CCT is expressed with an accelerated rate^26^, upregulation of lipid biosynthesis and adaptation are rendered futile. Thus the stress prevails and cells enter apoptosis. Importantly, the primary stress effects can be relieved by the heterologous expression of CCT within ~30 hours when cells have not yet entered the early apoptotic phase. Thus a transient transfection during the first 24 hours is expected to effectively revert the conditional CCT-knockout by efficiently restoring the phospholipid depletion.

Considering the devastating effect of the absence of a functional CCT on several organisms and cell lines, this inducible CCT deficient cell-based assay is an advantageous approach for studying CCT functionality with the conditional exclusion of the endogenous background compared to implementing knock-out of endogenous enzymes in the reporter cell line by recently emerged genome-editing techniques, such as CRISPR-Cas9, TALEN and ZFN^37,38^. However, this approach also has a number of limitations that are not yet resolved. The subcellular localization, the biomolecular interactions and turnover of the exogenous *Pf*CCT awaits to be thoroughly analyzed. A comparative evaluation of this model system and the infected red blood cells is additionally required e.g. with respect to the lipid composition differences and *Pf*CCT lipid-regulatory function. It is hitherto not known whether the native post-translational modification pattern of *Pf*CCT collected at PlasmoDB (gene ID: PF3D7_1133400)^39^ is also maintained within this heterologous expression system. Despite numerous studies, the effect of the conditional elimination of the endogenous CCT on the cellular machinery is still not completely explored. Further improvement and standardization of the methodology is also necessary e.g. to achieve higher transfection efficiencies. Nevertheless, our experimental data indicate that the herein described *in vitro* cell line model together with the redesigned modular *Pf*CCT cDNA presents a promising basis to establish a cell-based system for the functional analysis of *Pf*CCT. Moreover, as structural differences between the mammalian and protist CCT orthologs could enable development of *Plasmodium falciparum* specific CCT inhibitors, a cell-based test system with built-in comparison with the human CCT would not only be advantageous for structure-function studies but this model system may additionally be applied to screen effectiveness and toxicity of antimalarial therapeutic agents that target CCT within the PL biosynthesis pathway.

## Methods

### Materials

CHO-K1 and CHO-MT58 cells were a generous gift from Professor Dennis Vance (Department of Biochemistry, University of Alberta). Both cell lines were cultured in F-12 nutrient mixture (Ham) supplemented with 10% foetal bovine serum and 1% Penicillin-Streptomycin (Thermo Fisher Scientific, Waltham, MA, USA). Transient transfection was performed with FuGene HD transfection reagent (Promega, Madison, WI, USA). Trypsin-EDTA solution (TE) and Phosphate Buffered Saline (PBS) dissolved from PBS pH 7.4 powder (Merck KGaA, Darmstadt, Germany) were used for cell culture maintenance. For cellular experiments and cloning steps the plasmid pIRES-EGFP-puro (a gift from Michael McVoy (Virginia Commonwealth University), Addgene plasmid #45567) and a modified pBluescript SK (+) denoted as pBluescript SK (+)* was used (for details see Supplementary Information). The *E*. *coli* strain BL21 (DE3) Rosetta and pET15b plasmid was used for protein expression. Restriction enzymes, T4 DNA ligase and Phusion Hot Start Flex DNA polymerase were purchased from New England Biolabs (Ipswich, MA, USA). DNA purification kit was obtained from Macherey-Nagel (Düren, Germany). Isopropyl β-D-1-thiogalactopyranoside (IPTG) was obtained from Fisher Scientific GmbH (Schwerte, Germany). Nickel-nitrilotriacetic acid (Ni-NTA) was from Qiagen (Düsseldorf, Germany), protease inhibitor cocktail tablets were purchased from Roche (Basel, Switzerland). CTP, purine nucleoside phosphorylase and antibiotics were purchased from (Merck KGaA, Darmstadt, Germany). Phosphocholine chloride sodium salt hydrate (termed ChoP) was from TCI Europe N.V. (Antwerp, Belgium). MESG (7 methyl-6-thioguanosine) was obtained from Berry and Associates (Dexter, MI, USA). All other chemicals were of analytical grade of the highest purity available.

### Cloning of *Pf*CCT constructs

The *Pf*CCT 3D7 cDNA sequence (PlasmoDB: PF3D7_1316600) was codon-optimized for expression in *E*. *coli* (GenScript, Piscataway, NJ, USA). The *Pf*CCT_(528–795)_ H630N construct was obtained using the previously described *Pf*CCT_(528–795)_ (pET15b) second catalytic domain construct lacking the lysine-rich *Plasmodium* specific loop (720–737)^22^. For site-directed mutagenesis the QuikChange method (Agilent) was applied. Primer synthesis and verification of the mutagenesis was performed by Eurofins MWG GmbH. For cellular experiments constructs containing the full length *Pf*CCT sequence (*Pf*CCT_(1–896)_) were obtained. Due to the high sequence identity of the two catalytic domains of *Pf*CCT, resynthesis of the previously codon-optimized cDNA sequence was necessary (GenScript, Piscataway, NJ, USA) to install unique restriction sites for cloning and mutagenesis (for details see Supplementary Information). The *Pf*CCT_(1–896)_ was constructed in two sequential copy paste cloning steps by subcloning the full length sequence from (pUC57-Kan) to pBluescript SK (+)* using NheI/SacII restriction sites and from the cloning vector to pIRES-EGFP-puro using the NheI/XhoI restriction endonucleases. *Pf*CCT_(1–522)_ and *Pf*CCT_(523–896)_ were engineered by subcloning from the *Pf*CCT (pUC57-Kan) plasmid to pBluescript SK (+)* using the restriction enzyme pairs NheI/BamHI and BamHI/SacII, respectively. The inactivating (H/N) and thermosensitizing (R/H) mutations were introduced in the constructs harboring only one of the active sites by QuikChange mutagenesis (Agilent) using the forward and reverse primers H45N, H630N, R96H and R681H. The constructs *Pf*CCT_(1–896)_ H45N H630N and *Pf*CCT_(1–896)_ R96H R681H were cloned in the pBluescript SK (+)* plasmid using restriction enzymes NheI/BamHI/SacII and subcloned to the mammalian expression vector pIRES-EGFP-puro with NheI/XhoI restriction sites. Primer sequences are given in Supplementary Table 2. DNA sequences of the different constructs were verified by sequencing at Eurofins MWG GmbH or Microsynth AG.

### Protein expression and purification

The H630N point mutant variant of the His-tagged *Pf*CCT_(528–795)_ was expressed and purified as described previously^22^. The construct expressed in Rosetta (DE3)pLysS *E*. *coli* strain was induced at OD_600nm_ 0.4–0.6 with 0.5 mM IPTG for 18 h at 20 °C. To gain an appropriate purity of the protein Ni-NTA affinity chromatography was performed with 250 mM imidazole elution. The collected elution fractions were dialyzed into a buffer containing 20 mM HEPES 100 mM NaCl pH 7.5.

### Steady-state enzyme activity measurements

Steady-state activity measurements were performed as described previously^22^ with optimized modifications using a continuous coupled pyrophosphatase enzyme assay, which employs pyrophosphatase (PPase) and purine nucleoside phosphorylase (PNP) auxiliary enzymes and MESG substrate for colorimetric phosphate detection in concentrations 0.17 U/ml, 1.25 U/ml and 0.1 mM, respectively. During the titration CTP concentration was varied between 0 and 1.5 mM while ChoP concentration was kept at 5 mM. The *Pf*CCT_(528–795)_ H630N enzyme was used in 10 μM concentration and the slope of the absorbance change was determined after monitoring the reaction for 30 minutes. Kinetic data were fitted with Michaelis–Menten equation using OriginPro 8.6 (OriginLab, Northampton, Massachusetts, USA).

### Cell culture conditions

The CHO-K1 and CHO-MT58 cell lines were maintained in F-12 medium supplemented with 10% FBS and 1% Penicillin-Streptomycin at 37 °C or 40 °C in a humidified 5% CO_2_ atmosphere. Cell lines were regularly screened and the measurements were carried out on Mycoplasma-negative cells.

### Microscopy

CHO-K1 and CHO-MT58 cells grown in 6-well plates at 37 °C and 5% CO_2_ were transiently transfected with FuGene HD transfection reagent according the manufacturer’s instructions at a confluency of 80% with 2 μg purified plasmid DNA (*Pf*CCT_(1–896)_ in pIRES-EGFP-puro or empty vector of pIRES-EGFP-puro). After 24 h the incubation temperature was shifted to 40 °C in case of one set of cells. To ensure that the apoptosis starting between 30 and 48 hours is completed the transfected and non-transfected cells incubated both at 37 or 40 °C were inspected by fluorescence microscopy after 10 days. Brightfield or DIC and green fluorescent (EGFP) images were captured by a Leica DM IL LED 500 system using a Leica HCX PL Fluotar 40×/0.75 objective.

### Flow cytometry sample preparation and measurements

CHO-K1 and CHO-MT58 cells cultured in 6-well plates at 37 °C in a 5% CO_2_ environment were transiently transfected with FuGene HD transfection reagent according the manufacturer’s instructions at a confluency of 80% with 2 μg of purified plasmid DNA (*Pf*CCT_(1–896)_, *Pf*CCT_(1–896)_ H45N H630N and *Pf*CCT_(1-896)_ R96H R681H in pIRES-EGFP-puro). After 24 h the cells were treated with TE and the cell suspension was split 1:1 equally to two new plates, which were cultured at 40 °C and 37 °C, respectively. As cells are engaged to apoptosis within 30–48 hours CHO-K1 and CHO-MT58 cells were collected for flow cytometry analysis 72 hours after the transfection. After centrifugation at 1,100 rpm for 5 min (Eppendorf MiniSpin) the cells were resuspended in 600 μl PBS and were treated with propidium-iodide (PI) in a 1 μg/ml final concentration. The GFP positivity (%) was detected by FACS Attune^®^ Acoustic Focusing Cytometer, Blue/Violet (excitation wavelength: 488 nm solid state laser; emission filters: 530/15 nm). Intact cells were gated based on the forward scatter (FSC) and side scatter (SSC) parameters. Dead cells were excluded based on propidium-iodide positivity. The rescue potential of the different *Pf*CCT constructs was calculated based on the proportion of live (propidium-iodide negative) cells as follows:

1 $$Rescue\,potential\,( \% ):\frac{live}{all} \% \,of\,control=\frac{[(all-PI\,positive)/all]\,at\,40\,^\circ C\,}{[(all-PI\,positive)/all]\,at\,37\,^\circ C}\cdot 100$$

### Data availability

All data generated or analyzed during this study are included in this published article (and its supplementary information files).

## Electronic supplementary material

Supplementary Information (Marton, Vertessy)
