# Supplementary material for: Heterologous expression of CTP:phosphocholine cytidylyltransferase from Plasmodium falciparum rescues Chinese Hamster Ovary cells deficient in the Kennedy phosphatidylcholine biosynthesis pathway
Source: Sci Rep. 2018 Jun 12;8:8932. doi: 10.1038/s41598-018-27183-w (PMC5997628; doi:10.1038/s41598-018-27183-w)
Supplement: Supplementary file 1 — Supplementary Information (Marton, Vertessy) [file 41598_2018_27183_MOESM1_ESM.docx]

**Supplementary Information**

**Heterologous expression of CTP:phosphocholine cytidylyltransferase from *Plasmodium falciparum* rescues Chinese Hamster Ovary cells that are deficient in the Kennedy phosphatidylcholine biosynthesis pathway**

**Lívia Marton**^1,2,*^**, Fanni Hajdú**^1,3^**, Gergely N. Nagy**^1,3,†^**, Nóra Kucsma**^1^**, Gergely Szakács**^1,††^**, and Beáta G. Vértessy**^1,3,*^

^1^Institute of Enzymology, Research Centre for National Sciences, HAS, Budapest, 1117, Hungary

^2^Doctoral School of Multidisciplinary Medical Science, University of Szeged, Szeged, 6720, Hungary

^3^Department of Applied Biotechnology and Food Science, Budapest University of Technology and Economics, Budapest, 1111, Hungary

^†^Present address: Division of Structural Biology, University of Oxford, Roosevelt Drive, Oxford OX37BN, United Kingdom

^††^Present address: Institute of Cancer Research, Medical University Vienna, Vienna, Austria

[*marton.livia@ttk.mta.hu](mailto:*marton.livia@ttk.mta.hu), vertessy@mail.bme.hu

**Supplementary Figure 1** Alignment of CCT enzymes originating from different organisms and a related GCT. The first (*Pf*CCT_(1-210)_) and second (*Pf*CCT_(528-789)_) catalytic domain of *Plasmodium falciparum* CCT (Uniprot code Q8IEE9) is aligned to the extensively researched *Rattus norvegicus* CCT (*Rn*CCT, Uniprot code P19836), to the *Cricetulus griseus* CCT (*Cg*CCT, Uniprot code P49584) corresponding to the endogenous CCT of the thermosensitive CHO-MT58 cell line and to the related *Bacillus subtilis* GCT (*Bs*GCT, Uniprot code P27623). Conserved residues HxGH and R(Y/W)VD important in catalysis and dimer formation, respectively are denoted by black boxes. Strictly conserved residues are indicated by stars, whereas similar residues are depicted by colons or points.


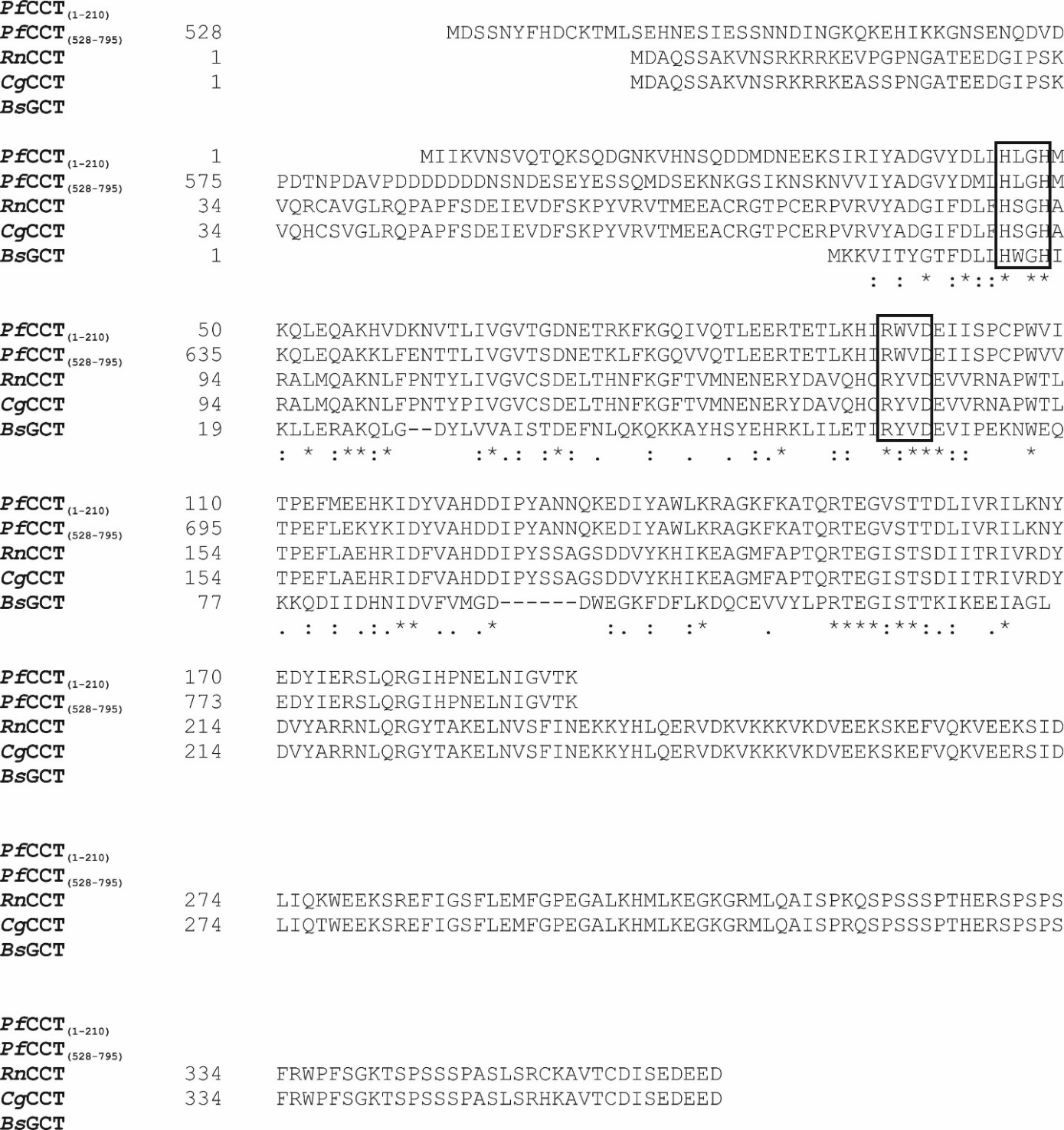


**Supplementary Figure 2** Multiple cloning site of the modified pBluescript SK (+)*


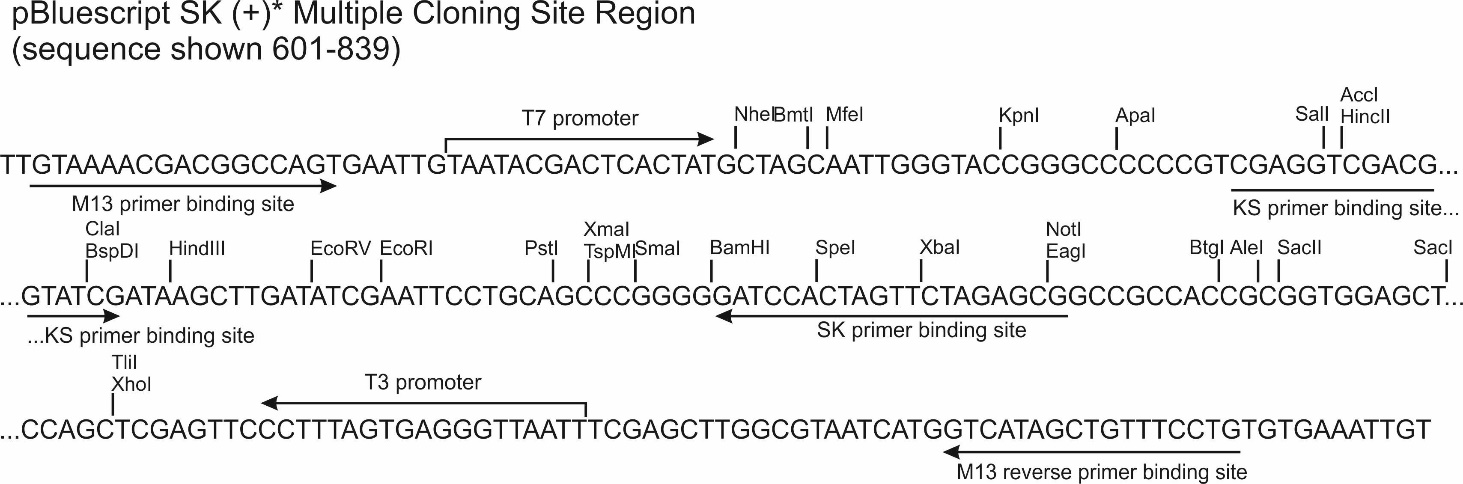


**Supplementary Figure 3** Construction of modular *Pf*CCT suitable for domain-specific site-directed mutagenesis. a) Nucleotide sequence of the redesigned *pfcct* gene. Note that nucleotide replacements due to the introduction of novel restriction sites are synonymous substitutions with a single exception of nucleotide exchange causing the Q524S residue replacement. Replaced nucleotides or amino acids are noted by bold and italic font style, and the inserted restriction sites are underlined. b) Negligible changes can be observed when comparing domain organization and predicted disorder profile (disorder prediction was performed by IUPred webserver^43^) of the wild-type *Pf*CCT and its redesigned form.


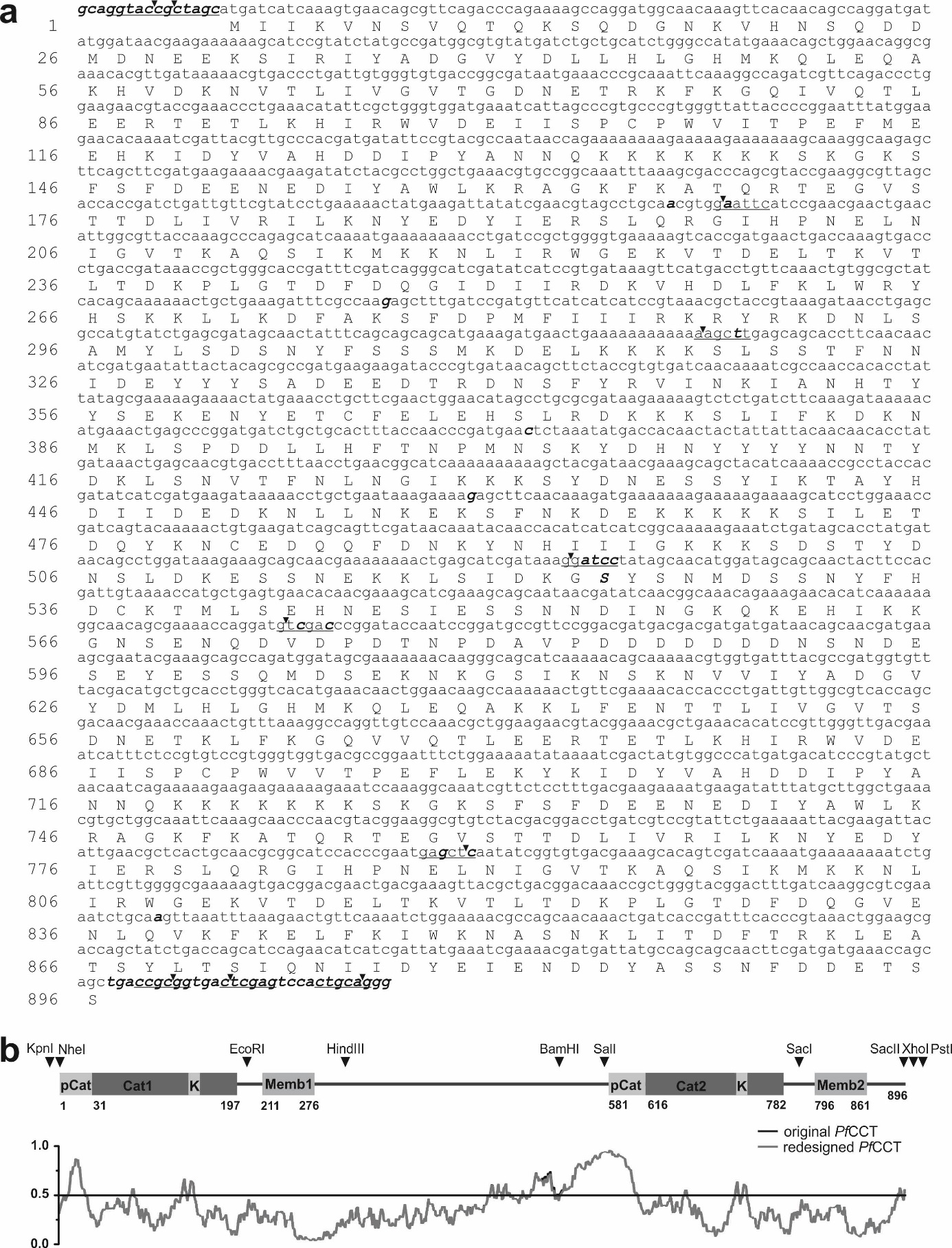


**Supplementary Table 1 Effect of *Pf*CCT protein variant overexpression on live cell fraction and rescue potential of CHO-K1 control cells.** Live fraction of cells was detected based on PI staining and intact cell detection. The same parameter as rescue potential was also determined for wild type CHO-K1 which does survive the temperature shift on their own. Flow cytometric data is a result of at least three independent experiments.

|  | **CHO-K1** | | |
| --- | --- | --- | --- |
| ***Pf*CCT protein variant** | **inactive** | **thermosensitive** | **wild type** |
| Live cell fraction (%) at 37 °C | 74.0 ± 4.6 | 81.4 ± 6.9 | 77.5 ± 8.7 |
| Live cell fraction (%) at 40°C | 48.0 ± 8.5 | 67.5 ± 11.7 | 59.2 ± 14.6 |
| **Rescue potential** | **65.4 ± 14.3** | **82.6 ± 8.1** | **76.0 ± 14.6** |

**Supplementary Table 2** Oligonucleotides used for cloning and mutagenesis

| **Primer name** | **5’-3’ sequence** |
| --- | --- |
| H45N for | GTGTATGATCTGCTGAATCTGGGCCATATG |
| H45N rev | CATATGGCCCAGATTCAGCAGATCATACAC |
| H630N for | GGTGTTTACGACATGCTGAACCTGGGT |
| H630N rev | GTTTCATGTGACCCAGGTTCAGCATGT |
| R96H for | CCCTGAAACATATTCACTGGGTGGATGAAATC |
| R96H rev | GATTTCATCCACCCAGTGAATATGTTTCAGGG |
| R681H for | GAAACACATCCATTGGGTTGA |
| R681H rev | GTCAACCCAATGGATGTGTTTC |
| pBs MCS1 for | CGACTCACTATGCTAGCAATTGGGTACCGGGCCCCCCGTCGAGGTCGAC |
| pBs MCS1 rev | GTCGACCTCGACGGGGGGCCCGGTACCCAATTGCTAGCATAGTGAGTCG |
| pBs MCS2 for | GTTCTAGAGCGAGCTCCACCGCGGTGCTCGAGCAGCTTTTGTTC |
| pBs MCS2 rev | GAACAAAAGCTGCTCGAGCACCGCGGTGGAGCTCGCTCTAGAAC |
